# Supplementary material for: Machine learning identifies pupil size and corneal thickness as key predictors of axial elongation rate
Source: PLoS One. 2026 Apr 24;21(4):e0348085. doi: 10.1371/journal.pone.0348085 (PMC13108789; doi:10.1371/journal.pone.0348085)
Supplement: Attachment — Submitted filename: REVIEWER COMMENTS 14 SEP 25.docx [file pone.0348085.s005.docx]

**Major Comments**

1. **Quantitative Analysis of CCT-Axial Length Relationship**
   The study identifies central corneal thickness (CCT) as a significant predictor but has not reported any quantitative analysis of this relationship. Could the authors apply their machine learning models to predict the specific correlation between each micron reduction in CCT and the corresponding increase in axial length?

This would provide clinically meaningful metrics for practitioners to assess myopia progression risk based on CCT measurements.

**2. Lens Thickness as a Predictive Factor**
Given that IOL Master measurements were obtained, Could the authors include lens thickness in their analysis and examine its relationship with axial length progression?

**Minor Comments**

**3. Examiner Standardization**
Please clarify whether ocular biometry and baseline refraction measurements were performed by a single optometrist or multiple assessors. If multiple assessors were involved, please provide information about inter-examiner reliability or standardization protocols to ensure measurement consistency across the study.

**4. Myopia Control Interventions**
The manuscript should explicitly state whether any study participants were using myopia control interventions (myopia control glasses, multifocal contact lenses, etc.) or were exclusively wearing single vision correction. This information is crucial for interpreting the progression rates observed in the study population.

**5. Age of Onset Definition**
The term "age of onset" requires clearer definition. Please specify whether this refers to: (a) the age at which the child was first diagnosed with myopia and prescribed corrective lenses, or (b) the age at which visual symptoms were first reported by the patient/parent. This clarification is important for reproducibility and clinical application of the findings.

**Suggested Addition**

**6. Mechanistic Discussion**
The authors should expand the discussion to include potential mechanisms underlying the association between thinner CCT and myopia progression. Consider discussing:

- Biomechanical properties of thinner corneas and their potential influence on scleral remodeling
- Possible correlations between corneal thickness and scleral thickness/strength
- Potential genetic factors that may simultaneously influence both corneal thickness and axial elongation

This mechanistic insight would strengthen the clinical relevance of the findings and provide a foundation for future research directions.

**Overall Assessment:** This study presents valuable insights into machine learning applications for myopia progression prediction. Addressing the above points would significantly enhance the manuscript's scientific rigor and clinical applicability while maintaining alignment with PLOS ONE's standards for reproducible research.
